# Genome-wide identification and evolution-profiling analysis of tps gene family in *Camphora longepaniculata* and screening of key TPS genes

**DOI:** 10.3389/fpls.2025.1546000

**Published:** 2025-02-28

**Authors:** Xin Liu, Yongkang Shuai, Xin Zhao, Minghu Zhang, Yue Yan, Jia Zhao, Ruizhang Feng, Qin Wei

**Affiliations:** ^1^ Sichuan Oil Cinnamon Engineering Technology Research Center, Yibin University, Yibin, Sichuan, China; ^2^ Faculty of Agriculture, Forestry and Food Engineering, Yibin University, Yibin, Sichuan, China

**Keywords:** *Camphora longepaniculata*, TPS gene family, evolution, gene duplicated events, expression profiles

## Abstract

*Camphora longepaniculata* is an important economic crop renowned for its rich volatile terpene compounds. Terpene synthases (TPS) are key enzymes in the biosynthesis of these compounds, playing significant roles in plant growth, development, and secondary metabolism. In this study, a total of 86 TPS genes were identified in *Camphora longepaniculata*, which were classified into five groups based on their evolutionary relationships. Analysis of cis-regulatory elements revealed associations between TPS genes and processes related to plant growth, development, and environmental stress responses. Gene Ontology (GO) enrichment analysis indicated that these TPS genes are predominantly linked to various enzymatic activities. Furthermore, analysis of duplication events revealed that tandem duplications (TD) and whole genome duplications (WGD) are major driving forces in the evolution of the TPS gene family. Notably, 18 TPS genes were found to be upregulated in high essential oil content varieties of *Camphora longepaniculata*. RT-qPCR validation further confirmed that *TPS26*, *TPS28*, and *TPS47* exhibit upregulated expression during leaf development, highlighting their potential involvement in terpene biosynthesis during this crucial developmental stage. These findings lay a solid foundation for further exploration of the functions of TPS genes in *Camphora longepaniculata*.

## Introduction

Terpenoids represent the largest and most diverse class of plant secondary metabolites, encompassing over 40,000 identified structures (Bohlmann and Keeling, 2008). These compounds play pivotal roles in plant growth, development, and ecological interactions (Loreto et al., 2014). For instance, Terpenoids act as phytoalexins in direct defense mechanisms and as signaling molecules in indirect defense responses, mediating interactions between herbivores and their natural enemies (Cheng et al., 2007). *TPS1* has been shown to enhance drought resistance in transgenic plants, making it a promising target for improving crop resilience (Yeo et al., 2000). Studies in other species, such as *Selaginella*, have revealed that functional TPS genes like *SlTPS1* contribute to heat and salt stress responses (Zentella et al., 1999). Their biosynthesis involves the sequential formation of C5 precursors, intermediate diphosphate derivatives, and a wide array of end products, with terpene synthases serving as the key catalytic enzymes (McGarvey and Croteau, 1995). Traditionally utilized in the food, pharmaceutical, and chemical industries, terpenoids are now gaining prominence for their applications in sustainable pest management and abiotic stress mitigation (Li et al., 2023a). Recent research have emphasized uncovering their ecological roles, characterizing biosynthetic pathways, and exploring their utility across various fields (Jahangeer et al., 2021). Additionally, advancements in metabolic engineering are enabling enhanced terpenoid production to meet industrial demands (Li et al., 2023a).

Terpenoids, also known as isoprenoids, are a highly diverse group of compounds derived from isoprene units (C5H8)n. They are classified based on the number of isoprene units they contain: hemiterpenes (C5), monoterpenes (C10), sesquiterpenes (C15), diterpenes (C20), triterpenes (C30), tetraterpenes (C40), and polyterpenes (Mabou and Yossa, 2021). these compounds play essential roles in both primary and secondary metabolism, exhibiting a wide range of biological activities, including anticancer and antimalarial properties. Terpene synthases (TPSs) are pivotal enzymes that catalyze the conversion of precursors such as geranyl diphosphate (GPP), farnesyl diphosphate (FDP), and geranylgeranyl diphosphate (GGDP) into a diverse array of mono-, sesqui-, and diterpenes (O’maille et al., 2008; Irmisch et al., 2012). Furthermore, other enzymes such as cytochrome P450s and transferases introduce additional modifications, significantly expanding the functional diversity of terpenoids. The biosynthesis of terpenoids involves intricate pathways that direct carbon flow and mediate cyclization reactions, enabling plants to produce hundreds of distinct terpenoids, with the total estimated in the thousands (Liu et al., 2018; Pichersky and Raguso, 2018). This remarkable diversity arises from unique biosynthetic mechanisms in plants that drive genetic and biochemical adaptations (Tholl, 2015). Terpenoids are indispensable for plant defense, interspecies communication, and the attraction of pollinators and seed dispersers, underscoring their critical role in ecological interactions. Their significance in sustainable pest management and abiotic stress tolerance has made them a focal point of recent research, highlighting their multifaceted contributions to plant ecology and resilience.

TPSs are essential enzymes that catalyze the biosynthesis of terpenoids, whose diversity is directly correlated with the number of TPS genes in plant genomes. Most plants possess approximately 20 to 152 TPS genes, with the notable exception of the moss *Physcomitrella patens*, which contains only a single functional TPS gene (Degenhardt et al., 2009). The TPS gene family is classified into two primary classes, I and II, and plays pivotal roles in plant metabolism, development, and responses to environmental stress. The expansion of this gene family, driven largely by segmental duplications, has significantly contributed to the functional diversification of TPS genes (Yang et al., 2012). Evolutionary analyses indicate that TPS genes likely originated from a common ancestor of land plants, with horizontal gene transfer events between microbes and plants potentially influencing their evolution (Chen et al., 2011). Structurally, plant TPS proteins possess both TPS and TPP domains, although the enzymatic activity of the TPP domain appears to have been lost over the course of evolution, likely serving a structural rather than catalytic role (Vandesteene et al., 2010; Yang et al., 2012). Furthermore, TPS genes exhibit tissue-specific and stress-responsive expression patterns, underscoring their functional specialization in plant development and adaptation (Xu et al., 2017). Phylogenetic studies have identified distinct subgroups within the TPS family, distinguishing genes involved in primary metabolism from those functioning in secondary metabolism (Whittington et al., 2002; Degenhardt et al., 2009). These findings offer valuable insights into the evolutionary history and functional divergence of the TPS gene family, enhancing our understanding of their roles in plant adaptation and survival.

*Camphora longepaniculata* (Gamble) Y. Yang, Bing Liu, and Zhi Yang is a culturally and economically significant evergreen tree species widely cultivated in the Yibin region of southwestern China. This species holds substantial economic value for its applications in the pharmaceutical, fragrance, and daily chemical industries (Wu et al., 2022). The leaves and twigs of *C. longepaniculata* are harvested primarily for essential oil extraction, which is rich in terpenoids, comprising over 85% of its composition (Hu et al., 2012). Major components of its essential oils include 1,8-cineole, α-terpineol, and γ-terpinene, known for their potent antibacterial, anti-inflammatory, and antioxidant properties (Wu et al., 2022). Among these, α-terpineol exhibits particularly strong antibacterial activity by disrupting cellular integrity (Li et al., 2012, 2014). The composition of essential oils varies significantly depending on factors such as growth environment, phenological stages, and plant varieties (Li et al., 2014). Furthermore, transcriptomic and metabolomic analyses suggest that variations in essential oil content and leaf phenotypes are linked to phenylalanine metabolism, specifically in flavonoid and terpenoid pathways (Zhao et al., 2022).

This study conducted a comprehensive genome-wide identification and characterization of the TPS gene family in *C. longepaniculata* based on the latest genomic information. A total of 86 CLTPS genes were identified. Detailed analyses included gene structure, motif composition, subcellular localization, protein 3D modeling, and expression profiling. The study examined the synteny of TPS genes among four *Lauraceae* species, as well as the role of cis-regulatory elements in the promoter regions during stress responses and developmental processes. Furthermore, transcriptome data analysis elucidated the expression patterns of TPS genes in *C. longepaniculata* varieties with high terpenoid content, providing valuable insights into the functional and evolutionary dynamics of the TPS gene family in this species.

## Materials and methods

### Plant materials and essential oil extraction

The *C. longepaniculata* plants were cultivated on the campus of Yibin University. In June, mature leaves were harvested, and essential oils were extracted using conventional steam distillation and a self-designed extraction still (**Supplementary Figure S1**) specifically developed for *C. longepaniculata* essential oil.

Traditional steam distillation method: 0.25 kg of dried *C. longepaniculata* leaves were weighed and subjected to distillation at 95.2°C for 120 minutes.

Self-developed essential oil extraction method: 2 kg of dried *C. longepaniculata* leaves were weighed, and heating temperatures were set to 105°C and 100°C, respectively. The stirring operation was maintained at 15.7 Hz, and distillation was carried out at 100°C for 100 minutes.

The oil yield can be calculated using the following formula:


$$Oil\;Yield\;\left(\%\right)=\frac{Weight\;of\;Essential\;Oil\;\left(g\right)}{Weight\;of\;Plant\;Materials\;\left(g\right)}$$

### Determination of 1-8-Cineole content in *C*. *longepaniculata* essential oil

The content of 1-8-Cineole in the essential oils extracted by the two methods was determined using Gas Chromatography (GC). The chromatographic conditions were as follows: the initial column temperature was set at 50°C and held for 10 minutes, then the temperature was increased at 4°C/min to 120°C, followed by an 8°C/min increase to 250°C, with a final 10-minute hold at 250°C. The injection port temperature was 270°C, and the injection volume was 0.1 μL. The column flow rate was 1.5 mL/min, with a split ratio of 100:1, and nitrogen with a purity greater than 99.999% was used as the carrier gas. The relative content of each chemical component in the *C. longepaniculata* essential oil was calculated using the area normalization method, where the area of each peak in the chromatogram was quantified relative to the total area to determine the concentration of 1-8-Cineole.

### Identification and chromosomal location of TPS gene family in *C. longepaniculata*

Genomic data and GFF file for *C. longepaniculata* (Yan et al., 2024) was downloaded from Figshare (https://figshare.com/s/ff6a0f810527f61ef63c), and for *C. chago* (Tao et al., 2024) from the National Genomics Data Center (accession number PRJCA022354; https://ngdc.cncb.ac.cn/gwh). The genomes of *C. kanehirae* (Chaw et al., 2019) (accession number PRJNA477266; https://www.ncbi.nlm.nih.gov) and *C. camphora* (Li et al., 2023b) (https://doi.org/10.6084/m9.figshare.20647452.v1) were also obtained. The typical domains of terpene synthases (TPS) were retrieved from the Pfam database (http://pfam.xfam.org/) under Pfam IDs PF01397 and PF03936. Hidden Markov model (HMM) searches were performed using HMMER 3.0 (Mistry et al., 2013), with an expected value (E-value) threshold set to 1e-5. Potential TPS protein sequences were further validated for TPS domain presence using the NCBI Conserved Domain Database (CDD; https://www.ncbi.nlm.nih.gov/cdd/), the SMART database (http://smart.embl-heidelberg.de/), and the Pfam database. Biochemical parameters of the identified TPS proteins were computed using the ExPASy online tool (https://web.expasy.org/protparam/). The subcellular localization of the TPS proteins was predicted using DeepLoc 2.1 (https://services.healthtech.dtu.dk/services/DeepLoc-2.1/).

### Phylogenetic relationship and conserved motifs analysis

The maximum likelihood (ML) method implemented in MEGA X (Kumar et al., 2018) was utilized to construct a phylogenetic tree for TPS proteins. The optimal model, LG+G, was determined using the “Find Best Protein Models” function in MEGA X. A bootstrap analysis with 1000 resampling iterations was performed to assess the robustness of the tree. The analysis included TPS proteins from *A. thaliana*, *C. longepaniculata*, *C. chago*, *C. kanehirae*, and *C. camphora*. The resulting phylogenetic tree file (*.nwk) was uploaded to the iTOL website (http://itol.embl.de/) for enhanced visualization. The conserved motifs of *C. longepaniculata* TPS proteins were analyzed using the MEME Suite (Bailey et al., 2009) (http://meme-suite.org/meme), with the maximum number of motifs set to 10.

### Analysis of promoter cis-acting elements

The 2000-bp upstream sequences of TPS genes from *C. longepaniculata*, *C. chago*, *C. kanehirae*, and *C. camphora* were employed to define the promoter regions. To identify potential cis-regulatory elements within these promoters, the Plant CARE database (http://bioinformatics.psb.ugent.be/webtools/plantcare/html/) was utilized. This database offers tools for the prediction and analysis of cis-regulatory elements, which are crucial for understanding gene regulation and expression patterns. The identified elements will be systematically classified and organized based on their functional categories, such as light responsiveness, hormone responsiveness, and stress-related elements. Following this classification, the results will be visualized using the ggplot2 package (Villanueva and Chen, 2019) in R.

Synteny analysis for TPS genesAnalyze the gene collinearity among *C. longepaniculata*, *C. chago*, *C. kanehirae*, and *C. camphora* based on their amino acid sequences and chromosome locations using MCScanX software (Wang et al., 2012a). The synteny circos plots was created using TBtools (v2.097) (Chen et al., 2023) and the Venn diagram was generated using EVenn (http://www.ehbio.com/test/venn/) (Yang et al., 2024) to show the genes that exhibit synteny with *C. longepaniculata* TPS genes across several species. Subsequently, we employed the approximate method implemented in KaKs Calculator version 1.2 (Zhang et al., 2006) to estimate the synonymous (Ks) and nonsynonymous (Ka) substitution rates of the orthologous genes among the four species. A Ka/Ks ratio greater than 1 indicates positive selection, a ratio of 1 indicates neutral selection, and a ratio less than 1 suggests negative selection. Identification and visualization of structural variations in the genomes of *C. longepaniculata* and *C. camphora* using GenomeSyn (Zhou et al., 2022).

### Prediction of protein pocket binding sites

The three-dimensional structures of the candidate *CLTPSs* in *C. longepaniculata* were predicted through Swiss-Model server (https://swissmodel.expasy.org/), which utilizes homology modeling techniques to generate reliable structural models based on known protein structures. Docking analysis of ligand-binding regions in the predicted protein models was performed using the P2Rank tool (https://prankweb.cz/) (Jakubec et al., 2022), followed by visualization in PyMOL (DeLano, 2002).

### Codon usage evaluation

Gene parameters, including total GC content (GC%), as well as GC1%, GC2%, GC3%, CAI, and ENC, were analyzed using CAICal (http://genomes.urv.cat/CAIcal/) (Puigbò et al., 2008). CAI evaluates the extent of translational selection shaping codon usage patterns and serves as a predictor for highly expressed genes (Sharp and Li, 1987). ENC, ranging from 20 to 61, quantifies codon usage bias, with a value of 20 indicating a single codon preference per amino acid and 61 signifying no codon bias (Fuglsang, 2004). Additionally, the relative synonymous codon usage (RSCU), calculated as the ratio between observed and expected frequencies of synonymous codons for each amino acid (Sharp and Li, 1987). To compare these gene parameters among group1, group2, group3, and group5 gene lineages across species, a Kruskal Wallis was conducted using the kruskal.test function in R. Statistical significance was evaluated, with * indicating a p-value< 0.01, ** indicating a p-value< 0.001, *** indicating a p-value< 0.0001, and **** indicating a p-value< 0.00001.

### Gene ontology

The TPS genes of *C. longepaniculata* were annotated using the eggNOG mapper v2 server (Huerta-Cepas et al., 2017; Cantalapiedra et al., 2021), which provides functional annotation based on evolutionary relationships and gene orthology. Following the annotation, Gene Ontology (GO) enrichment analysis is conducted using TBtools. The enrichment results will be classified according to molecular function (MF), cellular component (CC), and biological process (BP), selecting the top 20 entries from each category for visualization. The enrichment significance was determined based on a corrected p-value< 0.05.

### Prediction of protein networks

Using the STRING database (http://string-db.org/cgi), identify the protein-protein interaction (PPI) network of TPS genes based on orthologous genes between *C. longepaniculata* and *A. thaliana*.

### Expression patterns of *ClTPSs* in *C. longepaniculata* with high terpenoid content

Transcriptome data for various *C. longepaniculata* varieties exhibiting different terpenoid contents, including 1,8-cineole, terpineol, limonene, phytol, beta-myrcene, farnesal, and beta-sitosterol, were obtained from the Sequence Read Archive (SRA) database (https://www.ncbi.nlm.nih.gov/sra/) using the accession number PRJNA804339. The raw sequencing data were processed using Trimmomatic (Bolger et al., 2014), which effectively filtered out adapter sequences, low-quality reads, and unwanted contaminants to ensure data integrity. Key quality control steps included the removal of adapter sequences using Trimmomatic’s built-in Illumina adapter database, trimming low-quality bases from both ends of the reads, and applying a sliding window approach to remove regions with an average quality score below 15. Additionally, reads with a length shorter than 36 bp were discarded, ensuring only high-quality reads were retained. Subsequently, Kallisto (Bray et al., 2016) was employed for aligning the cleaned reads to the reference genome, followed by quantification of gene expression levels. This analysis yielded comprehensive expression data that reflect the transcriptional profiles of the different varieties. The transcript abundance was estimated by calculating FPKM (Fragments Per Kilobase of transcript per Million mapped reads). After quantification, the FPKM values were log-transformed to reduce the influence of highly abundant genes. These log-transformed FPKM values were then normalized to ensure consistency in gene expression levels. To visualize the overall gene expression patterns, heatmaps were generated using the R package ComplexHeatmap (Gu, 2022).

### RNA extraction and RT-qPCR validation

Leaves of *C. longepaniculata* plants grown at Yibin University were collected in February, April, and June, representing both the juvenile and mature leaf stages. For each time point, three biological replicates were collected. Total RNA was extracted from the leaves using the Plant RNA Extraction Kit-V1.5 (Chengdu Biofit Biotechnologies Co., Ltd., Chengdu, China). The concentration and purity of the RNA were measured using a NanoDrop 2000 (Thermo Fisher Scientific, Wilmington, DE).

Actin was used as the reference gene to normalize the relative expression levels, and quantitative real-time PCR (qPCR) was performed on a CFX96 PCR System. The 10 µl reaction mixture contained 5 µl of 2× TB Green Premix Ex Taq II (Tli RNaseH Plus), 0.4 µl of each primer (10 µM), 1 µl of cDNA, and sterile double-distilled water to a final volume of 10 µl. The qPCR amplification conditions were as follows: an initial denaturation at 95°C for 3 minutes, followed by 39 cycles of 95°C for 10 seconds and 58°C for 30 seconds. After the amplification, a melt curve analysis was conducted, starting at 95°C for 5 seconds, followed by a gradual temperature increase of 0.5°C/min from 65°C to 95°C. The RT-PCR primers were designed using Primer Premier Software (version 5.0), and the sequences are provided in **Supplementary Table S8**. A bar graph of expression levels was created using GraphPad Prism (v7.04). Statistical significance between each pair of groups was determined using one-way ANOVA, followed by multiple comparisons. Asterisks were used to indicate significant differences, with * denoting significance at p< 0.01, ** at p< 0.001, and *** at p< 0.0001.

## Results

### The content of essential oil and 1-8-Cineole in *C. longepaniculata* leaves were determined using two methods

Steam distillation for *C. longepaniculata* essential oil extraction exhibits suboptimal efficiency. We therefore engineered a continuous extraction apparatus to enhance productivity. Experimental results showed that the oil yield from steam distillation was 2.4%, while the oil yield using the newly developed extraction still was 2.83%, an increase of 17.92%. Additionally, the content of 1-8-Cineole (primary constituent) in the new method was 61.39%, compared to 51.76% with the traditional method, representing an 18.61% increase (**Table 1**).

**Table 1:** The content of essential oil and 1-8-Cineole in *C. longepaniculata* leaves were determined using two methods.

Method	Oil yield (%)	1-8-Cineole content (%)
Steam distillation method	2.4	51.76
Extraction still	2.83	61.39

### Characterization of TPS gene family

**Figure 1:**
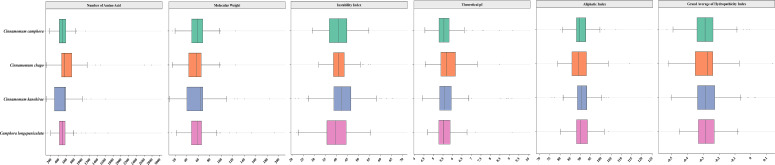
Physicochemical properties of TPS genes in four *Lauraceae* species. The X-axis represents various physicochemical properties of TPS proteins, including the number of amino acids, molecular weight, instability index, aliphatic index, theoretical pI and GRAVY, which can be used to assess their structural and functional characteristics. The Y-axis represents four *Lauraceae* species.

We performed genome-wide identification of TPS genes in four *Lauraceae* species using hidden Markov models (HMM) targeting conserved TPS domains (PF01397; PF03936). Our analysis identified 81 TPS genes in *C. camphora*, 72 in *C. chago*, 88 in *C. kanehirae*, and 86 in *C. longepaniculata*. The protein lengths of these TPS genes ranged from 79 to 278 amino acids, with isoelectric points varying from 4.41 to 9.81 and instability indices between 21.69 and 65.53. Except for *CchigoTPS52*, *CkTPS14*, and *CkTPS51*, the remaining TPS proteins exhibited hydrophilic characteristics (with a grand average of hydropathicity, GRAVY< 0) (**Figure 1**, **Supplementary Table S1**). Subcellular localization predictions showed cytoplasmic localization for 74.3% of TPS proteins, with 24.3% in plastids. Three TPS proteins localized to the endoplasmic reticulum and extracellular regions (**Supplementary Table S1**).

### Analysis of phylogenetic relationships of TPS gene family

**Figure 2:**
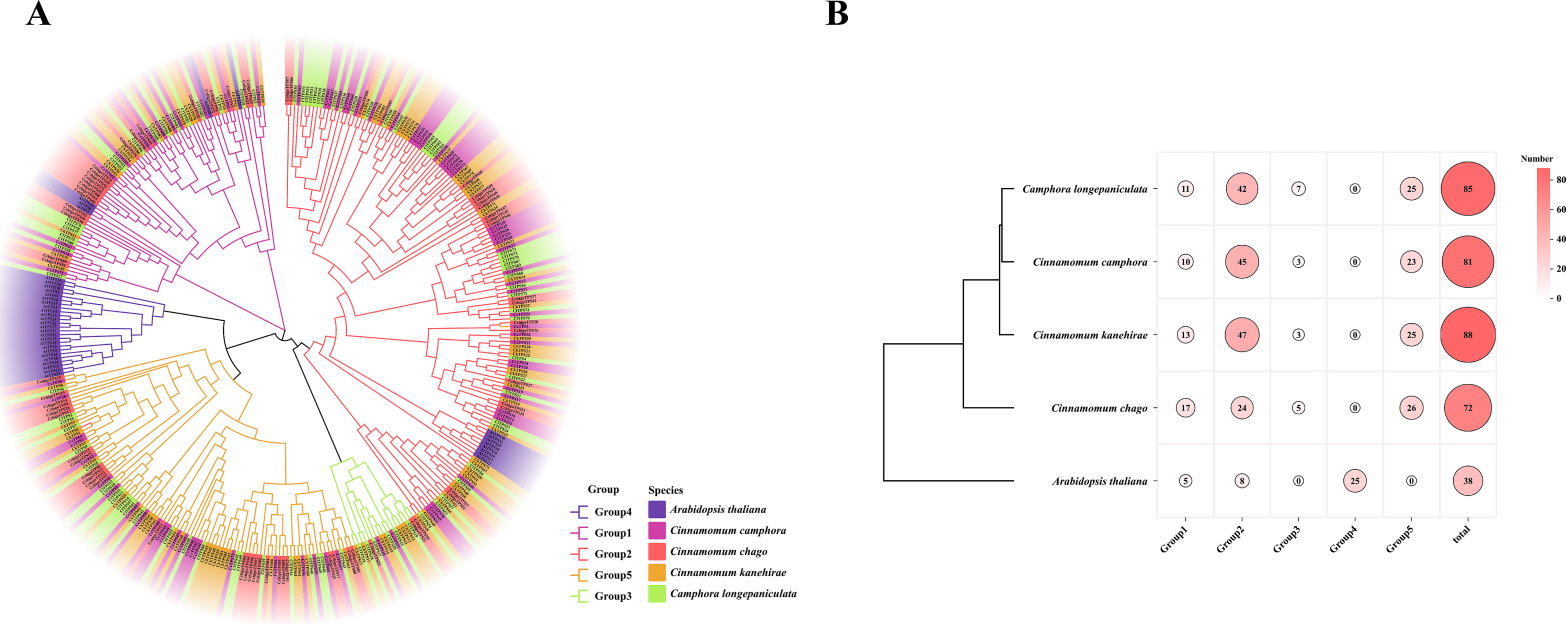
Phylogenetic tree of TPS genes in 5 species. **(A)** The evolutionary tree was constructed by the maximum likelihood method (bootstrap values: 1000 replicates) using MEGA X. **(B)** Statistical results of different subfamilies in different species.

A multiple sequence alignment of TPS sequences from four *Lauraceae* species and *A. thaliana* was performed using ClustalW to investigate the evolutionary relationships among TPS gene members. A phylogenetic tree was constructed based on the full-length amino acid sequences, revealing five distinct subfamilies (**Figure 2A**). The largest subfamily, Group 2, comprised 166 members, while Group 3 was the smallest, containing only 18 members. Group 4 exclusively contained *Arabidopsis* homologs. The distribution of TPS gene members among the four *Lauraceae* species exhibited similar patterns across the different subfamilies, though some variations were evident. In Group 2, three species (*C. camphora*, *C. kanehirae*, and *C. longepaniculata*) each harbored more than 40 members, whereas *C. chago* had fewer. showed maximum group 1 representation (n=17), contrasting with other species (**Figure 2B**). These findings highlight the evolutionary divergence of TPS genes within the *Lauraceae* family, as well as the distinct distribution patterns among species. These findings provide mechanistic insights into TPS functional diversification during terpenoid biosynthesis.

### Conserved motifs analysis of TPS gene family

To identify the conserved motifs of the TPS gene family in four *Lauraceae* species, the MEME suite was utilized to analyze the full-length protein sequences of TPS from each species. This analysis revealed a total of 10 distinct motifs (**Supplementary Figure S2**). The results indicated that *C. chago* exhibited 5 similar motifs compared to *C. longepaniculata*, while both *C. camphora* and *C. kanehirae* displayed 7 motifs that were similar to those in *C. longepaniculata*. Notably, four motifs (motif 1, motif 3, motif 4, and motif 7) were conserved across all four species, suggesting their potential significance in the evolutionary processes within the *Lauraceae* family. Interestingly, motif 8 was exclusively identified in *Cinnamomum camphora*, indicating unique evolutionary adaptations in this species. These findings enhance our understanding of the structural and functional diversity of TPS genes among *Lauraceae* species.

### Promoters analysis of TPS genes

**Figure 3:**
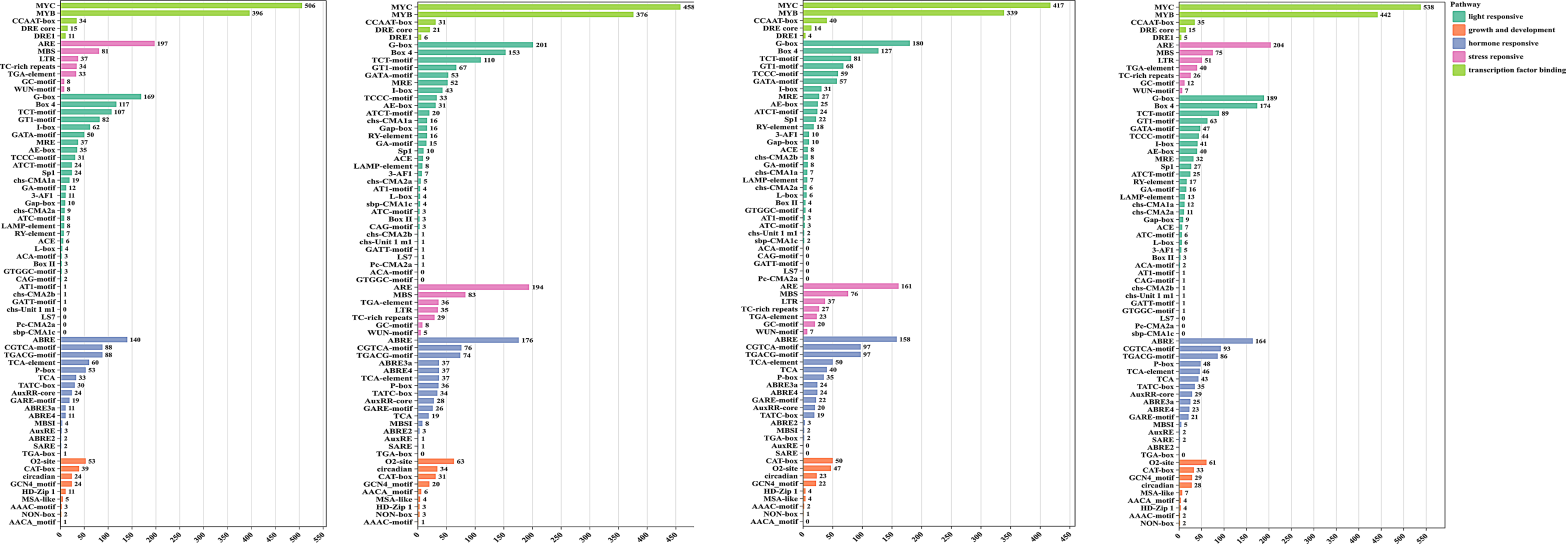
Analysis of cis-regulatory elements in the four species. Statistical summary and classification of the number of cis-regulatory elements in the four species. The X-axis represents the number of elements, and the Y-axis represents different types of elements.

To explore the transcriptional regulation and potential functional roles of TPS genes, we predicted the cis-regulatory elements in their promoter regions. Beyond the core cis-elements, we identified 69 distinct cis-acting elements in the 2-kb upstream region of the transcription start site of the TPS genes (**Supplementary Table S2**). These elements play critical roles in various biological processes, including stress responsive, hormone responsive, metabolic regulation, and growth and developmental processes (**Figure 3**). Transcription factor binding sites were the most abundant, followed by light-responsive and hormone-responsive elements (**Figure 3**). Except for *CcTPS36*, *CkTPS49*, and *CLTPS73*, all other TPS genes were found to contain at least one hormone-responsive element, the most prevalent of which was the abscisic acid response element (ABRE). Furthermore, the TPS promoter regions harbored several hormone-responsive elements, including auxin response elements (AuxRR core, TGA-element), MeJA response elements (CGTCA-motif, TGACG-motif), gibberellin response elements (TATC-box, P-box, GARE-motif), and salicylic acid response elements (TCA, SARE). Stress response elements were also found, including hypoxia (GC-motif), drought (MBS), wound (WUN-motif), low-temperature (LTR), and defense (TC-rich repeats).Among the four species analyzed, some genes were found to contain all types of cis-elements, with *C. longepaniculata* exhibiting 47, *C. camphora* exhibiting 68, *C. chago* exhibiting 54, and *C. kanehirae* exhibiting 75 genes (**Supplementary Table S2**). The findings suggest that TPS genes may play a role in the transcriptional control of plant growth, hormone regulation, and stress responses.

### Duplication events analysis of TPS genes

**Figure 4:**
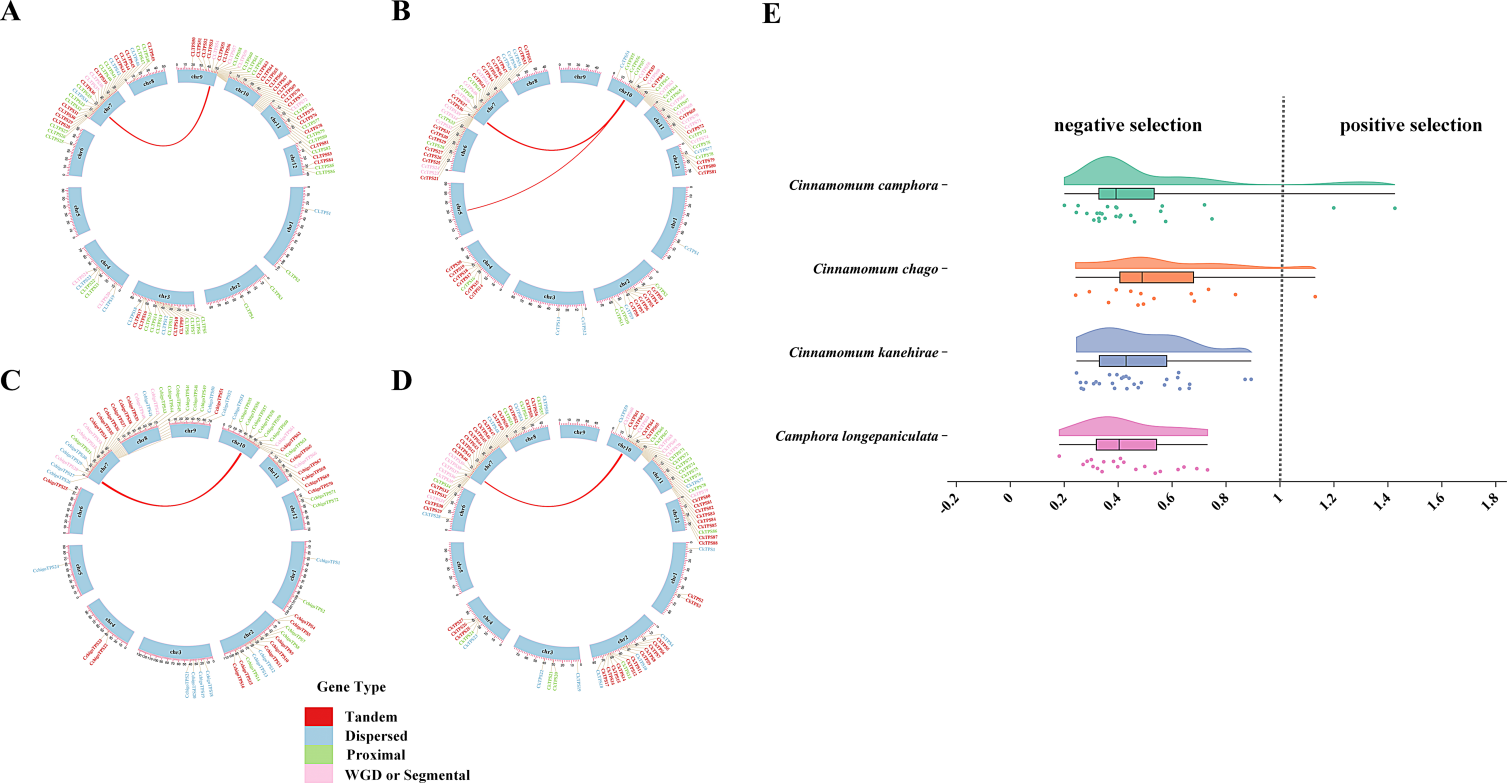
The duplicated gene pair and Ka/Ks values of TPS genes in 4 species. **(A)** *C. longepaniculata*, **(B)** *C. camphora*, **(C)** *C. chago*, **(D)** *C. kanehirae.* **(E)** The Ka/Ks values of TPS genes in 4 species.

This study extracted the physical locations of TPS genes based on the reference genome annotation files of *C. longepaniculata* and three other species within the *Lauraceae*. The results indicate that TPS genes are unevenly distributed across chromosomes, often in clusters. *C. longepaniculata* exhibits clustering of TPS genes mainly on chromosomes 3, 4, 7, and 9, whereas the other three species show clustering on chromosomes 2, 4, 7, and 10 (**Figures 4A–D**). This uneven distribution suggests a potential evolutionary adaptation of TPS genes within these species, highlighting the importance of genomic architecture in the regul.

Tandem duplications (TD) and whole-genome duplications (WGD) are significant drivers of gene family evolution and expansion. In this study, we analyzed the duplication events of TPS genes across four species. The results revealed 35, 35, 23, and 45 pairs of TD genes in species *C. longepaniculata*, *C. camphora*, *C. chago*, and *C. kanehirae*, respectively, along with 8, 16, 7, and 12 pairs of WGD genes (**Figures 4A–D**). These two types of duplicated genes account for 50.0%, 62.9%, 41.6%, and 64.7% of the total TPS genes in each species. This highlights the significant role of both TD and WGD in the amplification and diversification of TPS genes. To further investigate evolutionary pressures, we calculated the Ka, Ks, and Ka/Ks ratios for duplicated gene pairs using KaKs_Calculator. Most gene pairs displayed Ka/Ks ratios below 1 (**Figure 4E**), suggesting a predominant negative selection acting on these TPS genes, which may help maintain essential functions and prevent deleterious mutations. Conversely, the genes *CcTPS69*, *CcTPS70*, and *CchigoTPS4* demonstrated Ka/Ks ratios exceeding 1, implying they have undergone positive selection, potentially driving their diversification and adaptation to specific environmental pressures. This variation in selection pressures highlights the evolutionary dynamics within the TPS gene family.

### Collinearity analysis of TPS genes

**Figure 5:**
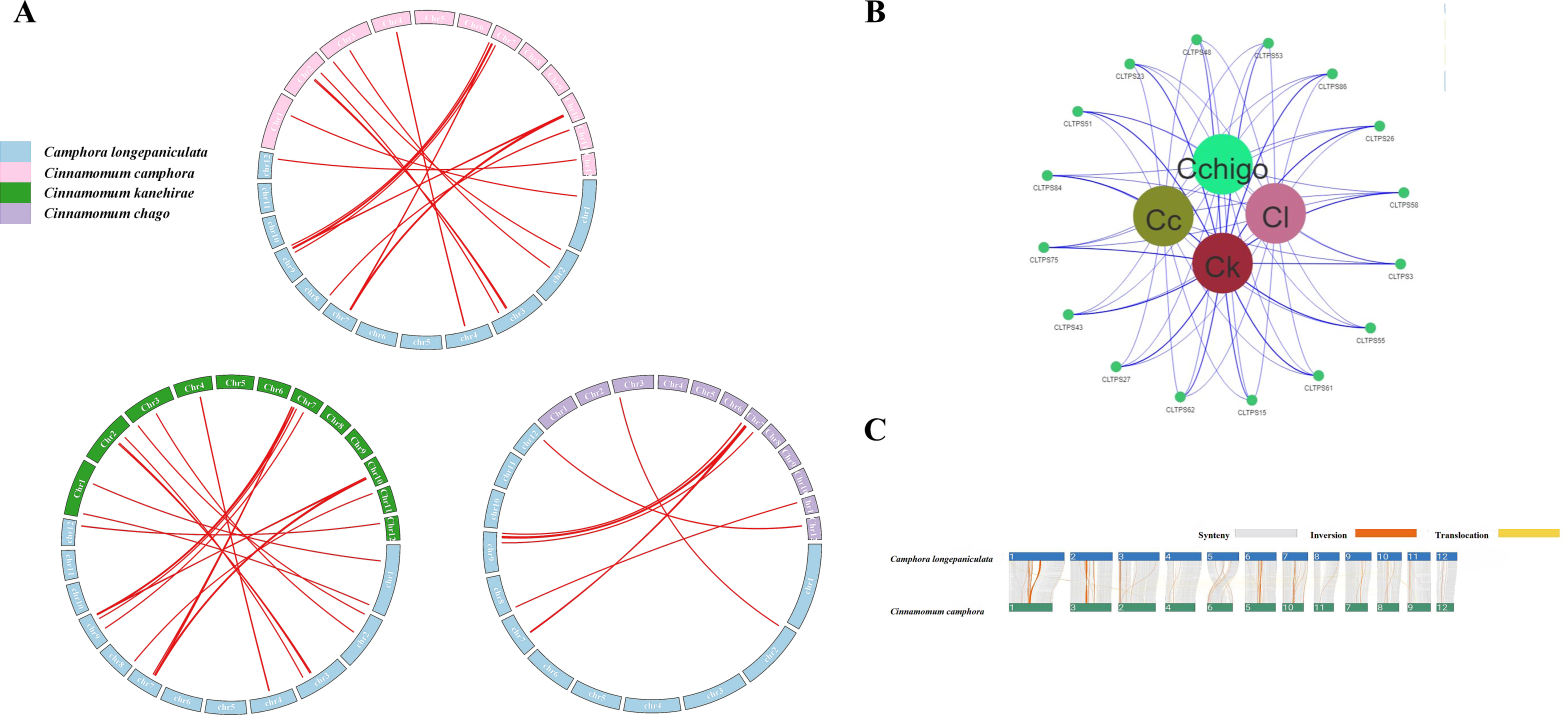
Syntenic relationships analysis of *TPS* genes in four *Lauraceae* species. **(A)** Syntenic relationships analysis of TPS genes between *C. longepaniculata* and three other *Lauraceae* species, **(B)** The collinearity relationship between four genomes, **(C)** Analysis of structural variations in *C. longepaniculata* and *C. camphora*.

To further investigate the evolutionary mechanisms of the TPS genes, we analyzed the homology relationships between *C. longepaniculata* and the other three species. The results indicated that *C. longepaniculata* shares 38, 36, and 42 direct homologous gene pairs with species *C. camphora*, *C. chago*, and *C. kanehirae*, respectively (**Figure 5A**). These homologous gene pairs are primarily concentrated on chromosomes 3 and 7 of *C. longepaniculata* (**Supplementary Table S3**), suggesting a potential genomic basis for the functional diversity observed within the TPS gene family across these species. Sixteen syntenic TPS genes showed pan-generic conservation. (**Figure 5B**) This conservation of specific TPS genes suggests their critical roles in maintaining similar functions or regulatory pathways throughout evolution. The presence of such conserved collinear genes underscores the importance of these TPS genes in the evolutionary dynamics of the *Lauraceae* family.

We also compared the structural variations between the genomes of *C. longepaniculata* and *C. camphora*, revealing a high degree of collinearity between the two genomes, accompanied by certain structural variations. The predominant type of variation observed was inversions, which were concentrated on chromosomes 1, 2, 6, and 7 (**Figure 5C**). Additionally, instances of translocations were noted, with specific segments on chromosomes 1 and 10 of *C. longepaniculata* corresponding to translocated regions on chromosomes 2 and 9 of *C. camphora* (**Figure 5C**). These findings indicate that while the overall genomic structure is conserved, rearrangements have occurred, which may influence the evolutionary trajectory and functional divergence of these species.

### Prediction of protein 3D structure

**Figure 6:**
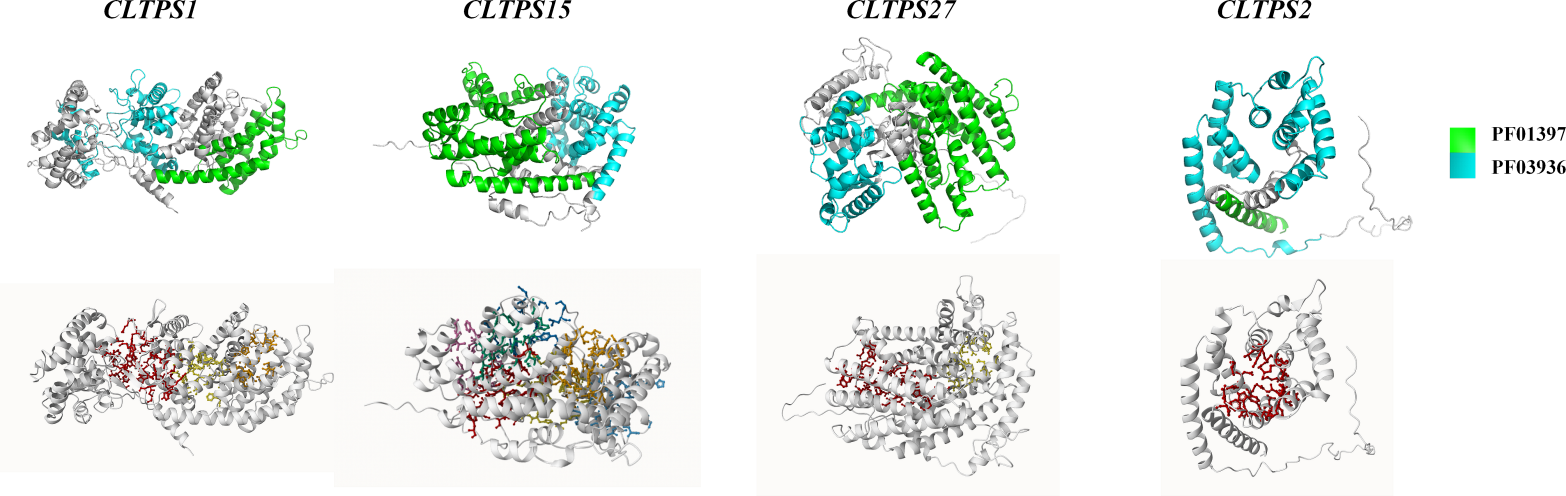
The 3D structure modeling and ligand binding site prediction in *CLTPSs.* One representative protein from each of the four groups was selected for modeling, with the proteins from left to right corresponding to *CLTPS1* (group 1), *CLTPS15* (group 2), *CLTPS27* (group 3), and *CLTPS2* (group 5).

For each of the four TPS gene groups in *C. longepaniculata* (noting that Group 4 has no members), we selected one TPS gene and conducted homology modeling of its three-dimensional structure using Swiss-Model (**Figure 6**). The analysis revealed that all four proteins predominantly feature α-helices as their main structural elements. The domains PF01397 and PF03936 were identified as key components of these TPS proteins, containing essential structures such as α-helices. We also predicted the protein binding pocket (**Figure 6**; **Supplementary Table S4**), where the surrounding amino acid residues are crucial in determining the physicochemical properties, conformation, and functionality of the proteins. These findings provide insights into the potential biochemical activities of the TPS proteins and their roles in terpenoid biosynthesis.

### Codon usage bias and other gene parameters

**Figure 7:**
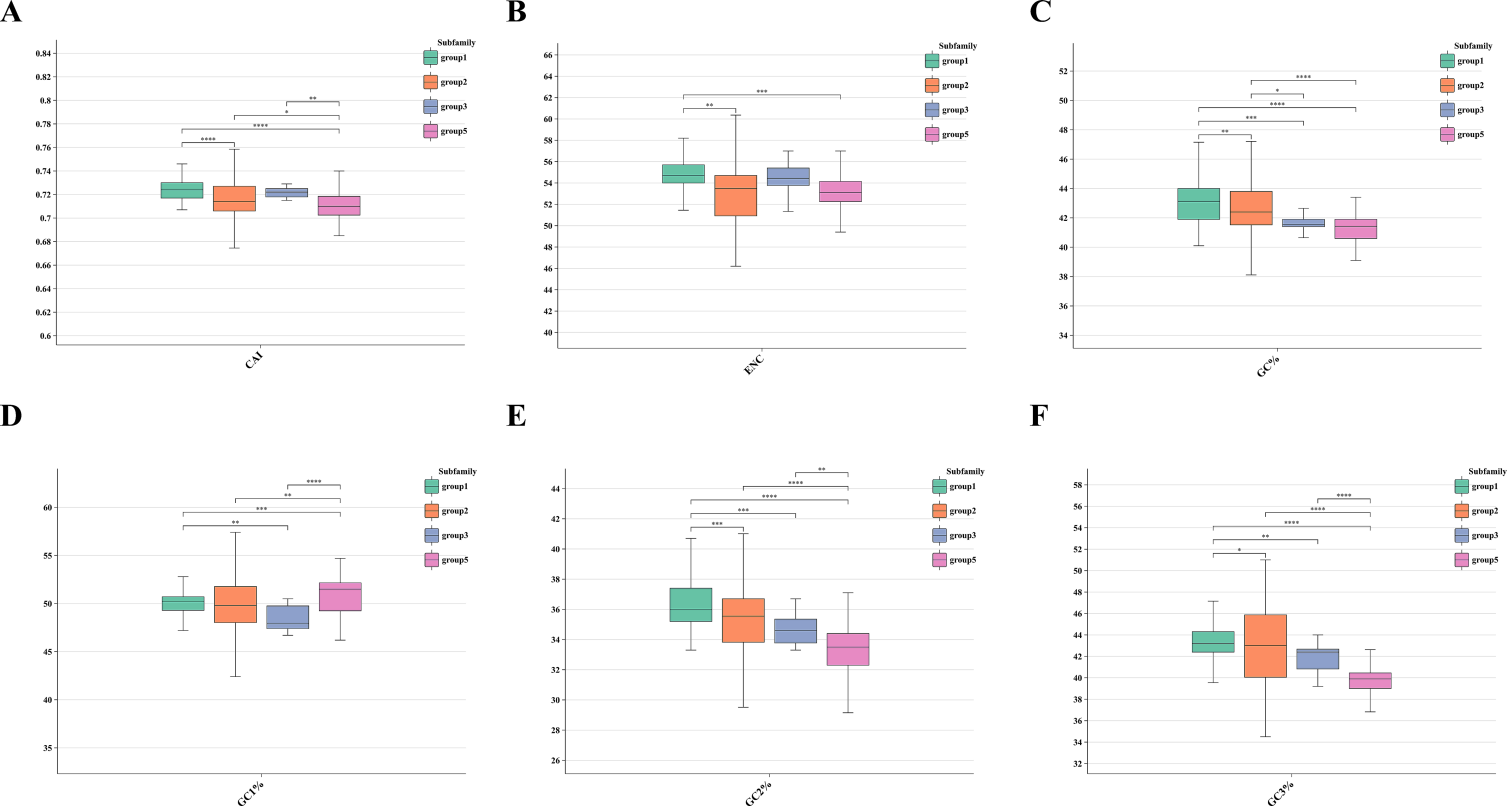
Comparison of gene parameters including the **(A)** CAI, **(B)** ENC, **(C)** total GC%, **(D)** GC1%, **(E)** GC2%, and **(F)** GC3% estimated for Group1, Group2, Group3, Group5 genes. The Y-axis represents the values of CAI and ENC, as well as the percentages of total GC%, GC1%, GC2%, and GC3%. Statistical significance was evaluated, with * indicating a p-value < 0.01, ** indicating a p-value < 0.001, *** indicating a p-value < 0.0001, and **** indicating a p-value < 0.00001.

We observed differentiated preferred codons and distinct gene characteristics among the four TPS gene lineages in the *Lauraceae* family (**Figure 7**; **Supplementary Figure S3**; **Supplementary Table S5**). For arginine (ARG), the codons AGA and AGG were the most frequently utilized across all lineages. Similarly, GGA was the preferred codon for glycine (GLY), and TCA was predominant for serine (SER). Notably, for proline (PRO), CCT was predominantly used in group 5, whereas CCA exhibited higher usage in groups 2 and 3 (**Supplementary Figure S3**). In addition, significant differences in gene parameters were observed among the various groups (**Supplementary Figure S4**). Group 1 and group 3 exhibited markedly higher codon adaptation index (CAI) and effective number of codons (ENC) compared to other groups. Meanwhile, the GC% of group 1 and group 2 was significantly greater than that of the other groups. group 5 displayed a distinct pattern, with higher GC1% and lower GC2% and GC3%. Strong pairwise correlations were observed between different gene parameters. For instance, CAI was positively correlated with overall GC% and GC3% but negatively correlated with GC1%. ENC showed a positive correlation with GC% and GC3%, while being negatively correlated with GC2%. These relationships suggest intricate codon usage biases and evolutionary constraints that likely influence TPS gene expression and function across the groups.

### Gene ontology functional annotation of *CLTPSs*

**Figure 8:**
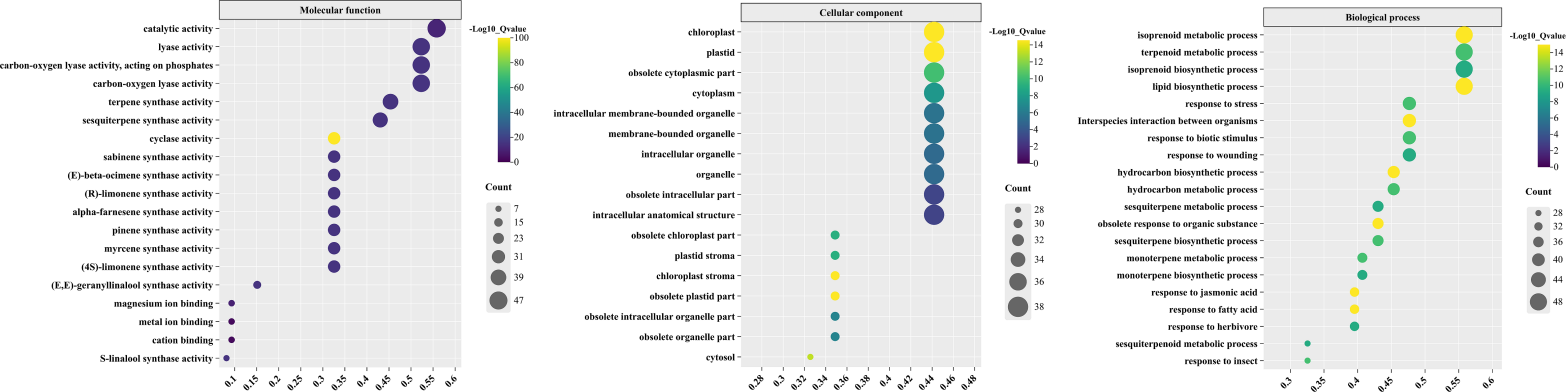
GO functional annotations of TPSs in *C. longepaniculata*. The X-axis represents the proportion of *CLTPS* genes in each GO term relative to the total number of *CLTPS* genes. The Y-axis lists the different GO terms, categorized into molecular function (MF), cellular component (CC), and biological process (BP).

To further explore the potential biological functions of TPS genes, we utilized the eggNOG-mapper database to perform Gene Ontology (GO) functional annotation on the TPS protein sequences from *C. longepaniculata*, followed by a comprehensive statistical analysis of these annotations (**Figure 8**; **Supplementary Table S6**).

The results indicated that, in the category of Molecular Function (MF), the TPS proteins are primarily associated with the activity of various enzymes, including cyclase, S-linalool synthase, terpene synthase, sesquiterpene synthase, and pinene synthase. In terms of Cellular Component (CC), the proteins are related to cellular structures, organelles, and chloroplasts. In the Biological Process (BP) category, the TPS genes are involved in the biosynthesis of metabolites and responses to stimuli. Notably, the key components of *C. longepaniculata* essential oil include various monoterpenols and terpenes, highlighting the significant role of TPS genes in the synthesis of these important phytochemicals. This functional characterization underscores the relevance of TPS genes in the biosynthesis of bioactive compounds that contribute to the plant’s ecological interactions and potential applications in pharmaceuticals and cosmetics.

### Prediction of protein networks

**Figure 9:**
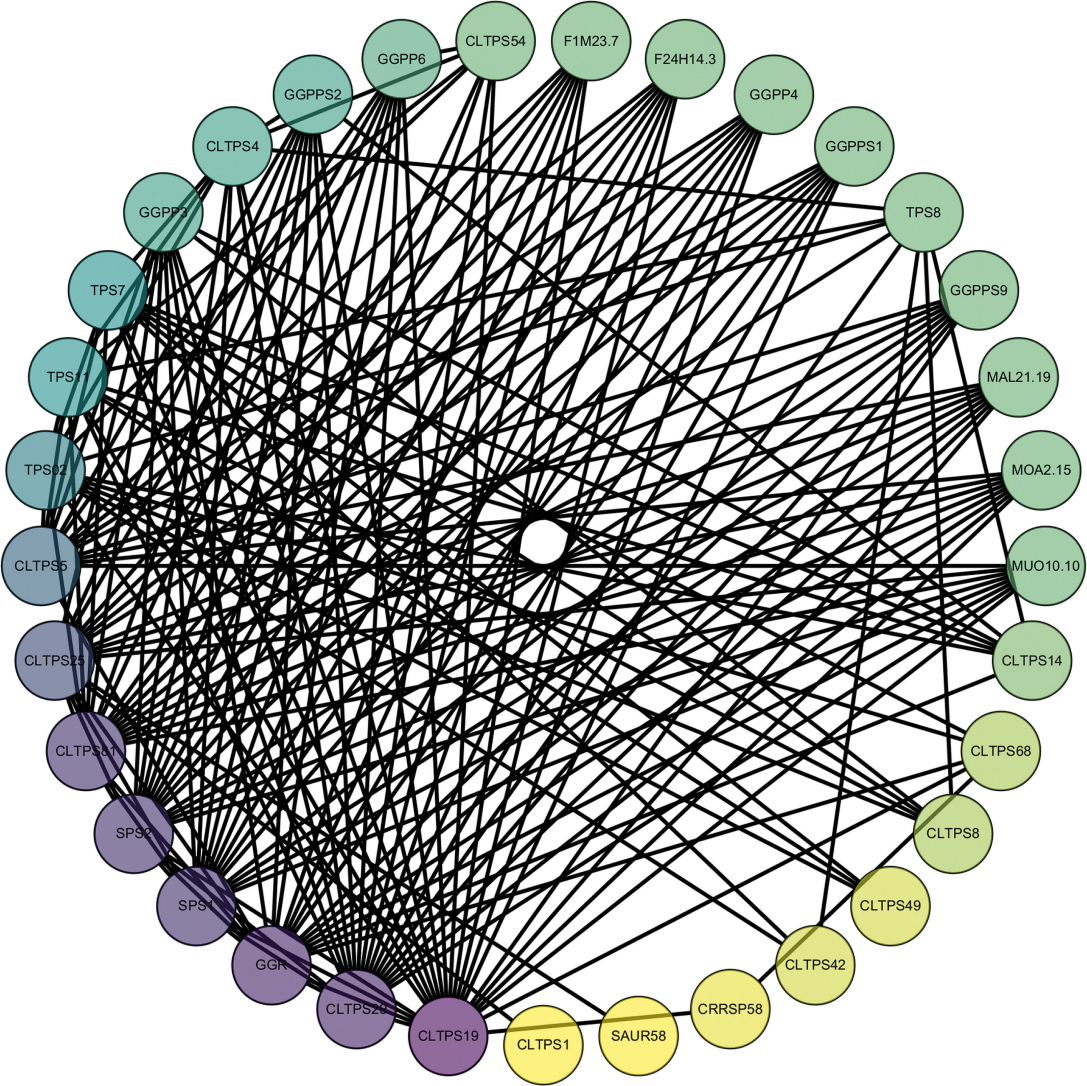
Protein interaction network predictions for TPS in *C. longepaniculata*. The colors range from yellow to purple, indicating the number of interacting proteins, from low to high.

To better understand the biological functions and regulatory networks of *CLTPSs*, we predicted their protein-protein interactions (PPIs) (**Figure 9**). *CLTPSs* interactors predominantly regulate secondary metabolite biosynthesis, particularly terpenoid, carbohydrate, and amino acid pathways. For example, genes like *GGPP3*, *GGPP4*, *GGPP6*, *GGPPS1*, *GGPPS2*, and *GGPPS9* are involved in the synthesis of isoprenoid compounds, particularly in the biosynthesis pathways of compounds like geraniol and other terpenoids (Zhang et al., 2021). These genes likely encode geranylgeranyl pyrophosphate synthases (*GGPPS*) and their homologs, enzymes critical in the synthesis of terpenoid precursors. The *SAUR* (Small Auxin-Upregulated RNA) proteins mediate auxin-regulated cell expansion and developmental responses (Ren and Gray, 2015). Moreover, Sucrose phosphate synthase (SPS) coordinates carbon allocation, influencing growth and stress adaptation (Li et al., 2024).

These findings suggest that TPS genes might play essential roles in regulating plant growth and development, response to environmental stresses, the synthesis of aromas and pigments, and the modulation of secondary metabolic pathways. This multifunctionality bridges primary and specialized metabolism, enhancing plant environmental adaptability.

### Expression analysis of *CLTPSs* in *C. longepaniculata* varieties with different essential oil contents

**Figure 10:**
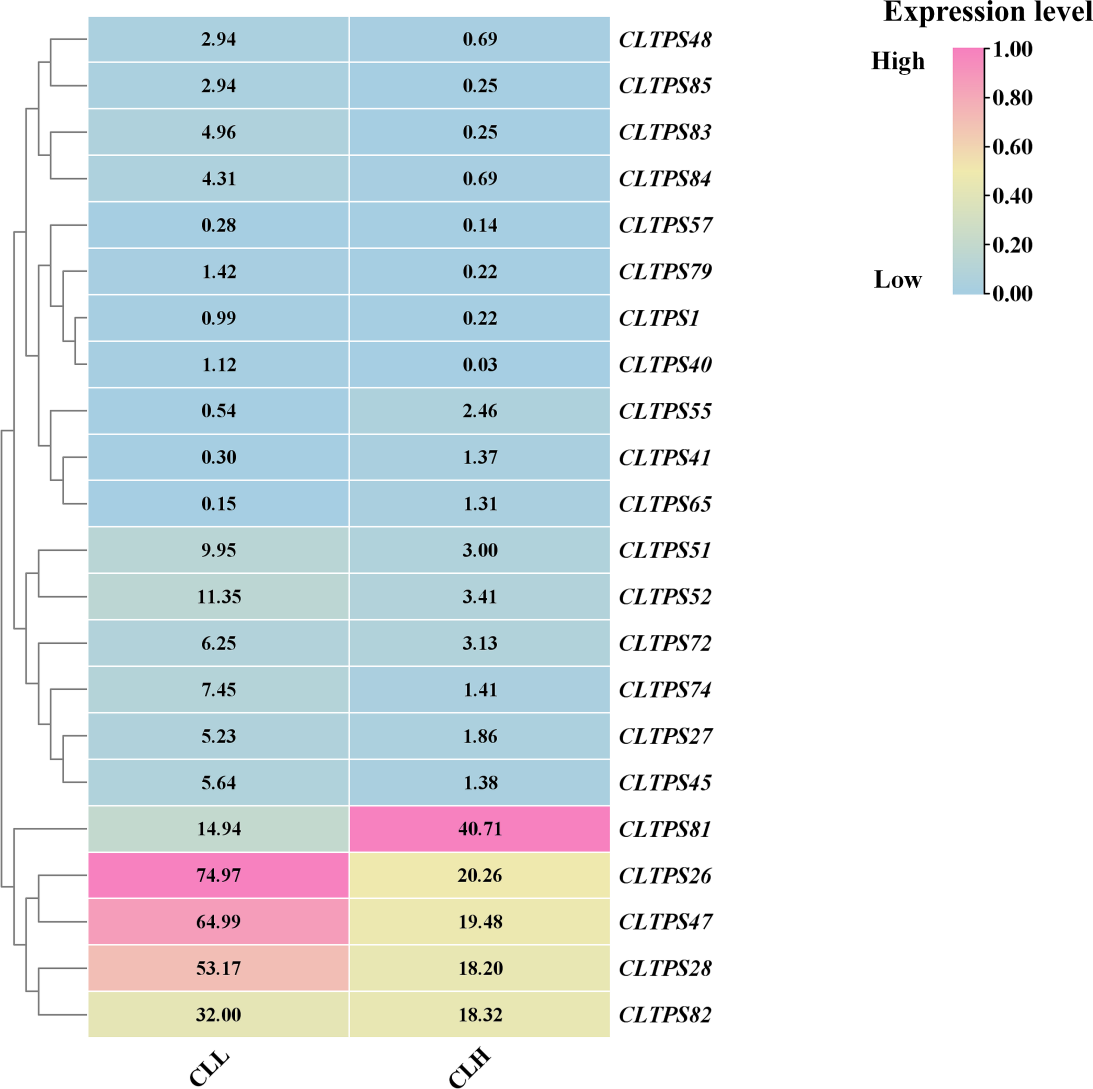
Gene expression analysis of TPS genes in in high essential oil content (CLL) and ordinary (CLH) varieties of *C. longepaniculata*. The heatmap illustrates the relative expression levels of TPS genes, with expression levels ranging from low (blue) to high (pink), providing a visual comparison between the two varieties.

To investigate the role of TPS genes in the production of essential oil in *C. longepaniculata*, we analyzed transcriptome data to examine the expression patterns of TPS genes in high essential oil content (CLL) and ordinary (CLH) varieties. A total of 22 differentially expressed TPS genes were identified between the two varieties, with 18 genes being upregulated in the CLL variety (**Figure 10**).This upregulation suggests that TPS genes play a significant role in the biosynthesis of essential oils, highlighting their importance in the high oil content phenotype of *C. longepaniculata*. These findings contribute to our understanding of the molecular mechanisms underlying essential oil production and may inform breeding strategies aimed at enhancing oil yield in this species.

### Validation of gene expression by RT-qPCR

**Figure 11:**
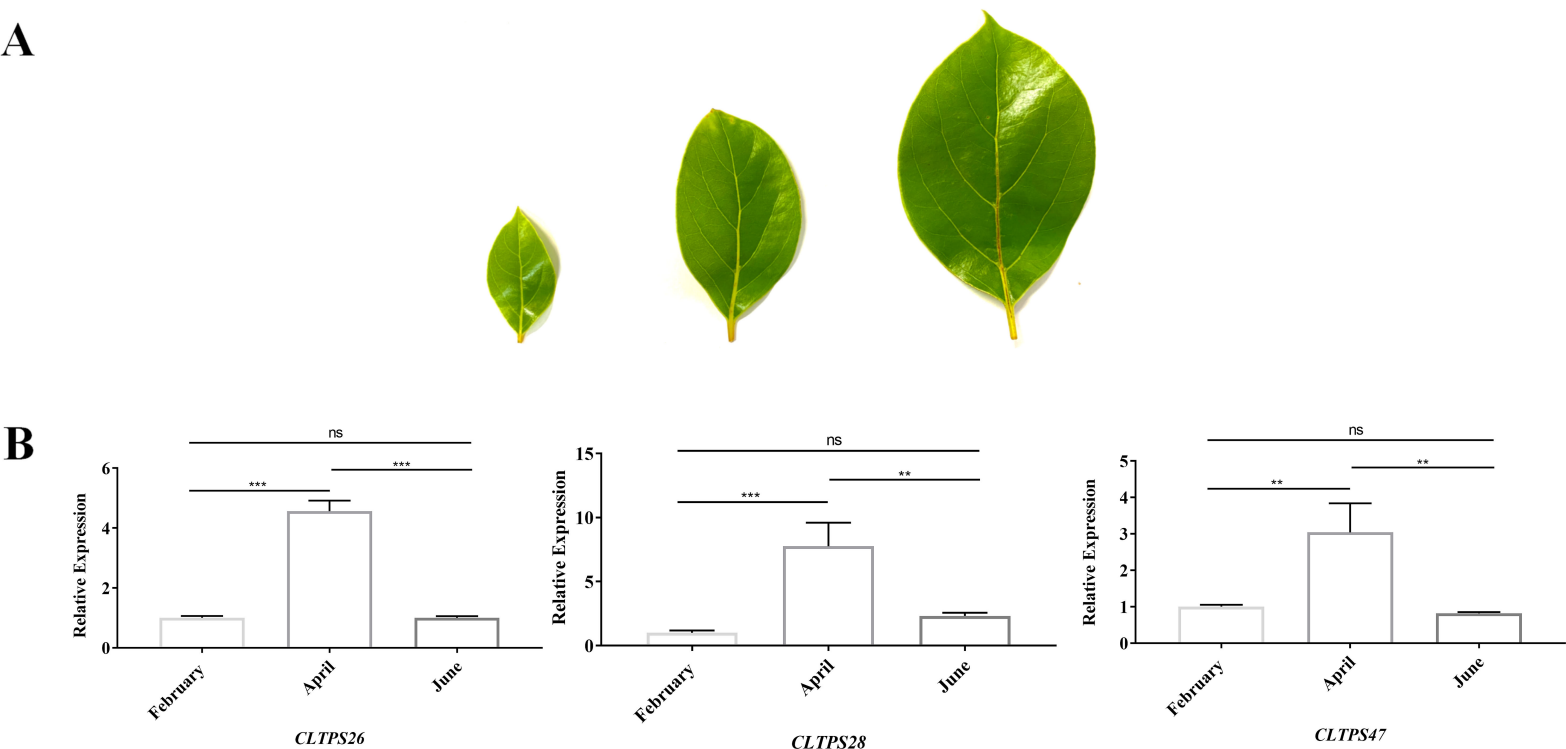
Morphological observation of *C. longepaniculata* leaves and TPS gene expression patterns. **(A)** Morphological characteristics of *C. longepaniculata* leaves observed in February, April, and June. **(B)** Expression levels of *CLTPS26*, *CLTPS28*, and *CLTPS47* across the three developmental stages. Asterisks were used to indicate significant differences, with * denoting significance at p< 0.01, ** at p< 0.001, and *** at p< 0.0001.

To investigate the expression levels of TPS genes at different developmental stages of *C. longepaniculata* leaves, samples collected in February, April, and June were analyzed. Three genes, *CLTPS26*, *CLTPS28*, and *CLTPS47*, which exhibited the highest expression levels in transcriptome data, were selected for this study. The results showed that all three genes reached their peak expression levels in April, followed by a decline in June (**Figures 11A, B**). Notably, while *CLTPS47* exhibited its lowest expression in June, the other two genes showed the lowest expression levels in February. These findings suggest that the expression levels of TPS genes follow a trend of increasing and then decreasing during leaf development, which aligns with the observed changes in terpenoid compound content, indicating a potential role for TPS genes in terpenoid accumulation.

## Discussion

Yibin is the largest base for *C. longepaniculata* in China, with a cultivation area of up to 630,000 acres. The annual production of *Cinnamomum* essential oil exceeds 14,000t, with 1,8-cineole being the primary component, constituting one-third of the global supply. This compound is widely used in food, cosmetics, and pharmaceuticals (Li et al., 2014). Currently, the main method for extracting *C. longepaniculata* essential oil is batch steam distillation, which is energy-intensive, highly polluting, and labor-costly, leading to significant waste of *C. longepaniculata* resources. In contrast, the self-designed extraction still used in this study allows for continuous production and improves the oil yield by 17.92% compared to traditional methods. This enhancement has the potential to significantly boost the economic benefits of the *C. longepaniculata* industry.

TPS genes mediate plant adaptation to abiotic stresses through specialized metabolite biosynthesis.

Beyond stress adaptation, the silencing of *BtTPS* via RNAi in transgenic plants has proven effective in controlling pests like whiteflies (Gong et al., 2022). Similarly, the terpene-rich essential oil of *C. longepaniculata*, dominated by 1,8-cineole and α-pinene, has demonstrated insecticidal activity against agricultural pests, highlighting its potential as a natural pesticide source. In maize, the TPS-b class gene *ZmTPS14* is induced upon feeding by herbivores such as the *Ostrinia furnacalis* and *Holotrichia parallela* larvae, suggesting its role in herbivore defense mechanisms (Sun et al., 2023). In rice, *OsTPS18* encodes a sesquiterpene synthase that produces (E)-nerolidol, an antibacterial compound that protects against bacterial pathogens (Kiryu et al., 2018). Additionally, infestation by *Chilo suppressalis* larvae led to the identification of 25 differentially expressed *OsTPS* genes, many of which are involved in the biosynthesis of diterpene phytoalexins and the ent-kaurene biosynthesis pathway (Sun et al., 2022). Such strategies could be integrated into integrated pest management (IPM) programs, promoting sustainable agriculture.

TPS are crucial enzymes responsible for the diversity of terpenes. They catalyze the conversion of isoprenyl diphosphates into various forms, including volatile monoterpenes, sesquiterpenes, semi-volatile compounds, and non-volatile diterpenes (Gutiérrez-del-Río et al., 2021; Li et al., 2023a). The synthesis and accumulation of a wide variety of volatile terpenoids in these plants can significantly alter the quality and flavor of the oils, presenting substantial commercial value for oil-producing plants (Yan et al., 2023). This diversity in terpenoid production not only has implications for enhancing the sensory qualities of essential oils but also opens avenues for the selective breeding of oil-producing plants. By targeting key TPS genes involved in terpenoid synthesis, breeders could improve both the yield and the specific chemical profile of oils, catering to specialized markets in food, cosmetics, and pharmaceuticals. This study comprehensively identified 86 TPS genes in *C. longepaniculata* and investigated their phylogenetic relationships, gene duplication events, cis-regulatory elements in promoter sequences, and expression patterns of the TPS gene family in *C. longepaniculata* with varying essential oil contents. These findings enhance our understanding of the molecular mechanisms governing terpenoid biosynthesis and their implications for plant resilience and essential oil production. The diverse terpene profiles facilitated by TPS gene diversity also offer promising opportunities for breeding programs aimed at enhancing oil quality and flavor in terpenoid-rich plants.

Phylogenetic analysis clustering revealed five subfamilies (group1 to group 5), with group 2 containing more than 50% of *Lauraceae* TPS genes and group 5 exclusive to *A. thaliana*. Among the 10 identified motifs, 5 were present in all four *Lauraceae* species, with both *C. camphora*, and *C. kanehirae* sharing 7 similar motifs with *C. longepaniculata*. This indicates a significant level of conservation and similarity in the TPS gene family across these species.

Cis-regulatory elements within plant TPS genes play a crucial role in mediating responses to environmental stress and signaling pathways during growth and development, and they have been extensively studied across various plant species (Yadav et al., 2020; Zhu et al., 2024). In addition to core cis-elements, We identified 69 cis-regulatory elements in 2-kb promoter regions. TPS genes coordinate terpenoid biosynthesis with developmental and stress-adaptation processes. These include plant growth and development, where they may influence developmental pathways; stress responses, where they contribute to the plant’s ability to cope with adverse conditions such as drought, salinity, or temperature extremes; and light signaling, which is crucial for optimizing photosynthesis and energy use. The presence of multiple hormone-responsive elements indicates that TPS genes are part of complex regulatory networks, regulating metabolic pathways in response to both internal and external signals. Additionally, GO enrichment analysis results also validate this point, highlighting the involvement of TPS genes in various biological processes, including metabolic pathways, stress responses, and developmental regulation. These findings provide a theoretical basis for improving abiotic stress resistance in economically important trees (such as avocado and cinnamon) through precise regulation of genetic elements mediated by CRISPR/Cas9. This multifaceted role underscores the importance of TPS genes in enhancing plant resilience and adaptability in changing environments.

WGD and small-scale duplication events duplications drive functional innovation in plant genomes (Wang et al., 2012b). In the four analyzed species of the *Lauraceae* family, the duplicated TPS genes accounted for 50.0% (*C. longepaniculata*), 62.9% (*C. camphora*), 41.6% (*C. chago*), and 64.7% (*C. kanehirae*) of the total TPS gene complement, respectively. This substantial representation of duplicated genes contributes significantly to the uneven distribution of TPS genes across different chromosomes. It is generally accepted that, due to selective pressures, amino acid sequences with important biological functions exhibit relatively low variability among different species (Delph and Kelly, 2014). Most TPS genes have undergone strong negative selection, indicating that these genes are relatively conserved. This conservation is essential for maintaining the functional integrity of terpenoid biosynthesis pathways, which are critical for plant defense, communication, and adaptation to environmental stresses. Additionally, the prevalence of WGD-derived TPS duplicates in *Lauraceae* suggests that polyploidy events have played a key role in enhancing terpenoid biosynthesis, which could be further harnessed in breeding programs to improve the resilience and commercial value of oil-producing plants.

*C. longepaniculata*, native to the Yibin region of Sichuan in Southwest China, is an economically significant tree species with considerable local importance. Various parts of the plant are utilized for essential oil extraction (Wu et al., 2022). Key components of the leaf essential oil include 1,8-cineole, α-pinene, and γ-terpinene, which are highly regarded for their excellent antibacterial, anti-inflammatory, and antioxidant properties (Li et al., 2014). Comparative transcriptomics revealed 18 TPS genes upregulated in high-oil genotypes (CLL) versus controls(CLH). This suggests the potential for TPS genes to be targeted in genetic improvement efforts aimed at enhancing the economic value of *C. longepaniculata* through increased essential oil production.

## Data Availability

Publicly available datasets were analyzed in this study. This data can be found here: Download the genome data of C. longepaniculata from the Figshare database(https://figshare.com/s/ff6a0f810527f61ef63c). C. chago from the National Genomics Data Center (accession number PRJCA022354; https://ngdc.cncb.ac.cn/gwh). C. kanehirae from the National Genomics Data Center (accession number PRJNA477266; https://www.ncbi.nlm.nih.gov). C. camphora from the Figshare database (https://doi.org/10.6084/m9.figshare.20647452.v1). Transcriptome data for various C. longepaniculata varieties exhibiting different terpenoid contents were obtained from the Sequence Read Archive (SRA) database (https://www.ncbi.nlm.nih.gov/sra/) using the accession number PRJNA804339.
