# Supplementary material for: Genome-wide identification and evolution-profiling analysis of tps gene family in Camphora longepaniculata and screening of key TPS genes
Source: Front Plant Sci. 2025 Feb 28;16:1546000. doi: 10.3389/fpls.2025.1546000 (PMC11906476; doi:10.3389/fpls.2025.1546000)

**Figure S1** The 100 L *C. longepaniculata* essential oil extraction kettle is made of stainless steel. The kettle features external insulation, internal stirring, a spiral feeding mechanism, and bottom electric heating. The system is equipped with a vacuum system and a glass condenser for assistance. Centralized control is used for operation, with the total power of the equipment being approximately 3 kW and the voltage set at 220 V.


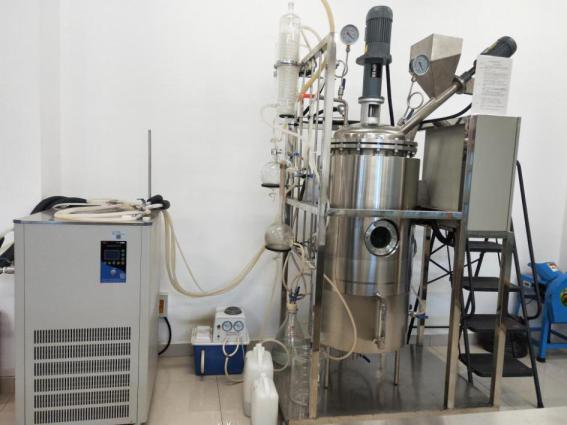


**Figure S2** The conserved motifs among the four species are presented from left to right as follows: *C. longepaniculatum*, *C.chago*, *C. kanehirae*, and *C. camphora*. Boxes of the same color indicate similar motifs.


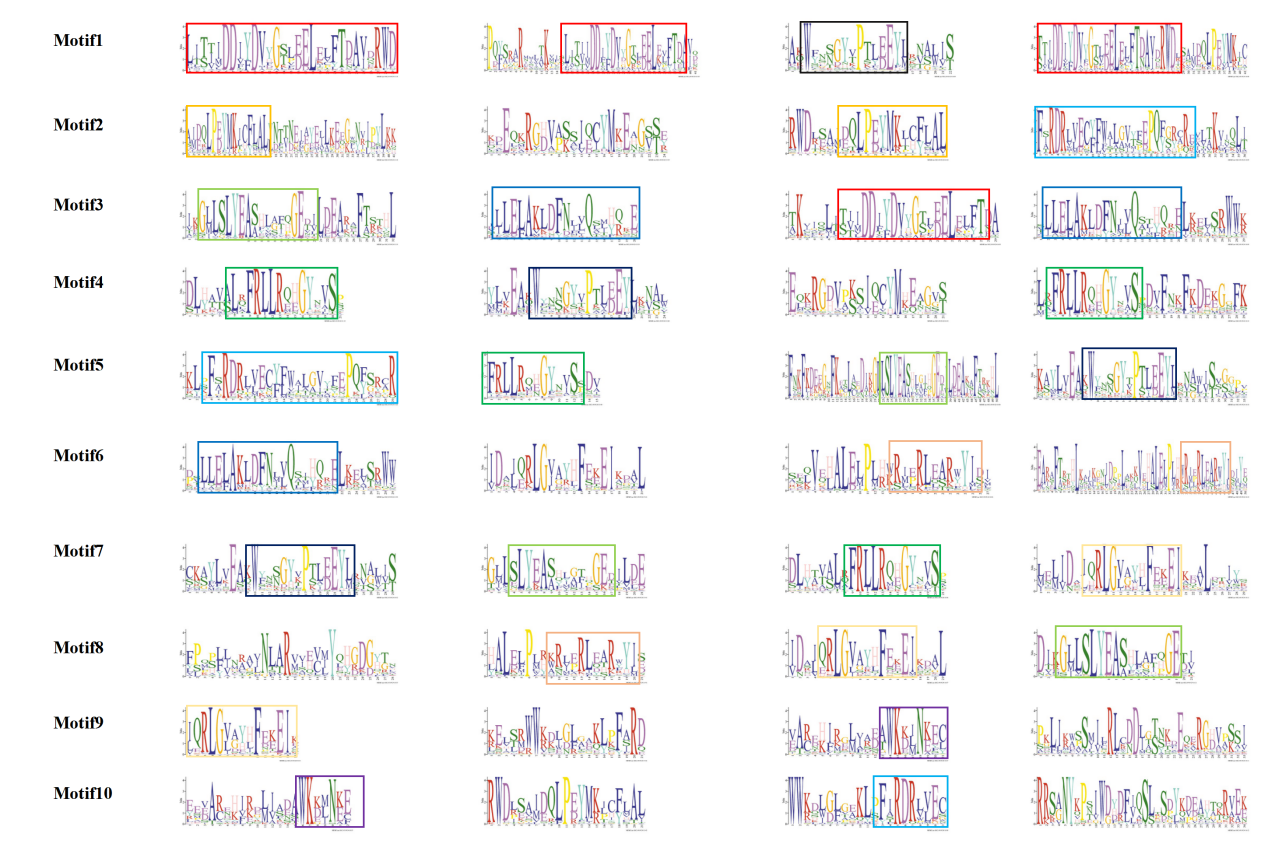


**Figure S3** Figure S6. Heatmap of average RSCU scores estimated for different codons in four Lauraceae TPS gene lineages. Average codon usage below 1.5 are colored from white to blue, while codons with RSCU values larger than 1.5 are colored with red.


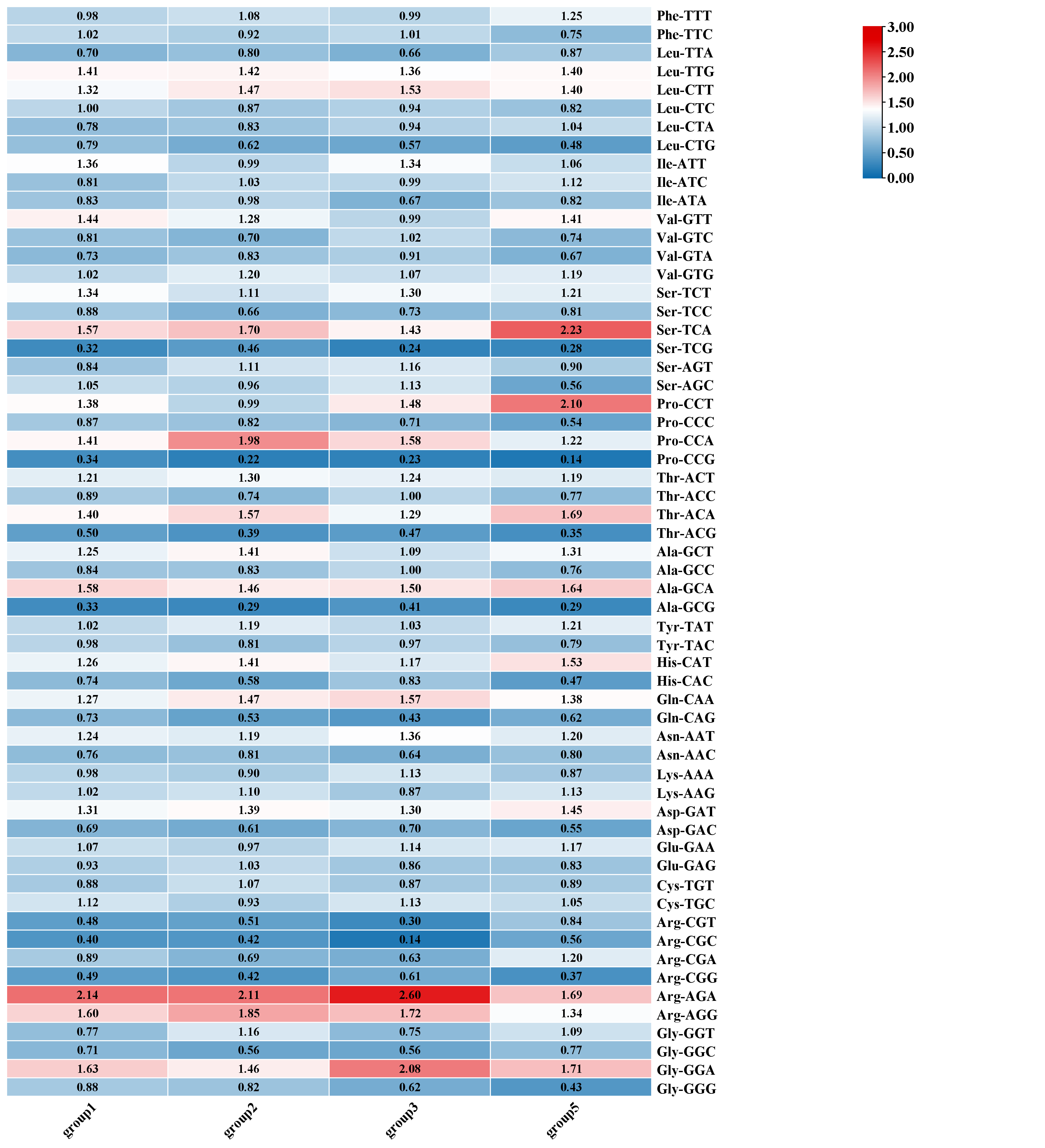


**Figure S4** The Pearson correlation among the estimated gene parameters of TPS genes in four *Lauraceae* species was calculated. * indicates a significant correlation at the p < 0.05 level, while ** indicates a highly significant correlation at the p < 0.01 level.


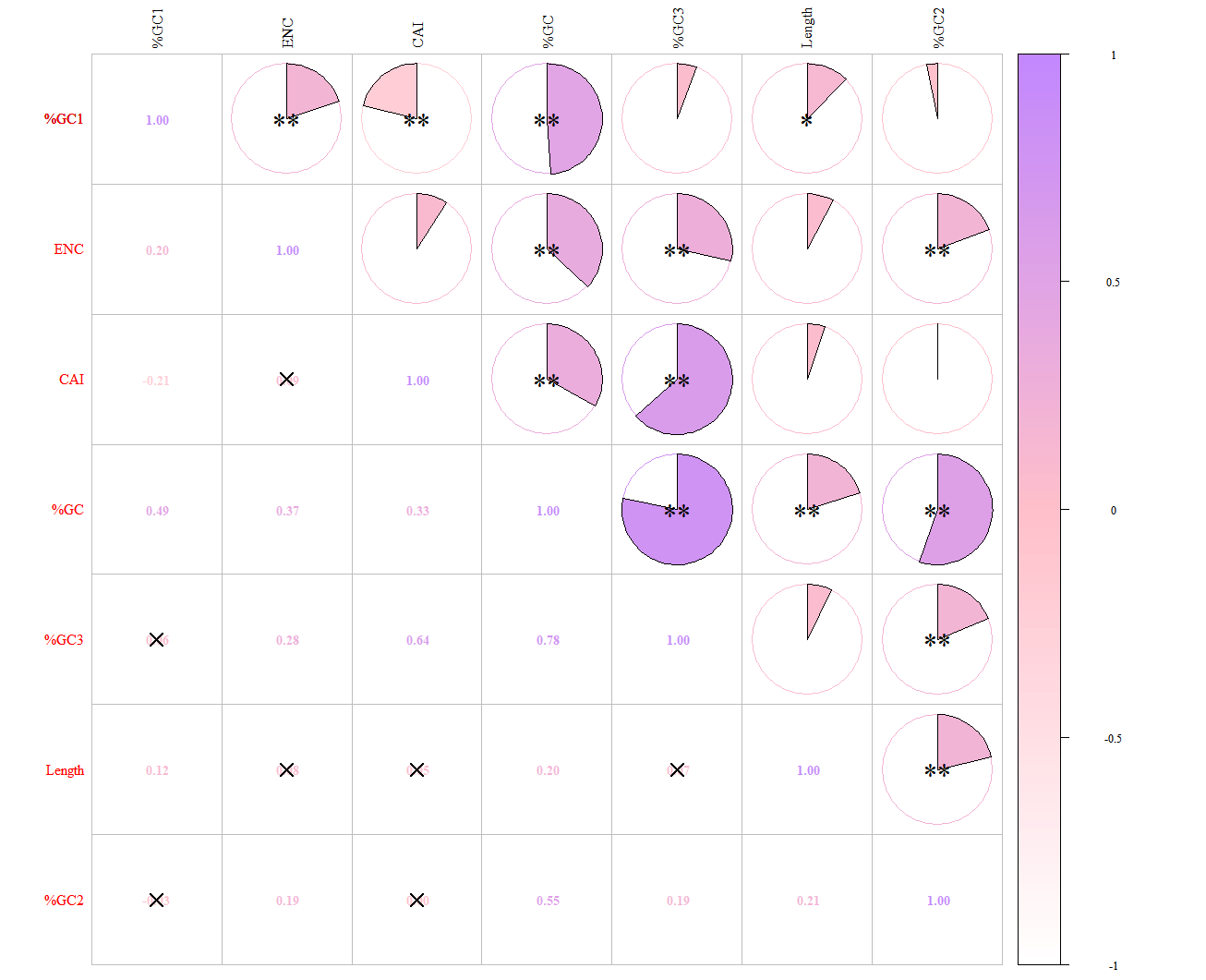

Supplement: Supplementary file 1 [file DataSheet1.docx]
